# PP2A methylesterase PME‐1 suppresses anoikis and is associated with therapy relapse of *PTEN* ‐deficient prostate cancers

**DOI:** 10.1002/1878-0261.13353

**Published:** 2023-04-18

**Authors:** Anna Aakula, Aleksi Isomursu, Christian Rupp, Andrew Erickson, Nikhil Gupta, Otto Kauko, Pragya Shah, Artur Padzik, Yuba Raj Pokharel, Amanpreet Kaur, Song‐Ping Li, Lloyd Trotman, Pekka Taimen, Antti Rannikko, Jan Lammerding, Ilkka Paatero, Tuomas Mirtti, Johanna Ivaska, Jukka Westermarck

**Affiliations:** ^1^ Turku Bioscience Centre University of Turku and Åbo Akademi University Finland; ^2^ HUSLAB Laboratory Services, Helsinki University Hospital Medicum and Institute for Molecular Medicine Finland FIMM University of Helsinki Finland; ^3^ Weill Institute for Cell and Molecular Biology & Meinig School of Biomedical Engineering Cornell University Ithaca NY USA; ^4^ Institute of Biomedicine University of Turku Finland; ^5^ Cold Spring Harbor Laboratory NY USA; ^6^ Department of Pathology Turku University Hospital Finland; ^7^ Department of Urology Helsinki University Central Hospital Finland; ^8^ Department of Life Technology University of Turku Finland; ^9^ InFLAMES Research Flagship Center University of Turku Finland; ^10^ Foundation for the Finnish Cancer Institute Helsinki Finland; ^11^ Present address: Faculty of Life Science and Biotechnology South Asian University New Delhi India; ^12^ Present address: The school of Life Science and Biopharmaceutics Shenyang Pharmaceutical University China

**Keywords:** anoikis, integrin, LAP2A/B, nuclear lamina, PP2A‐C methylation, PPME1

## Abstract

While organ‐confined prostate cancer (PCa) is mostly therapeutically manageable, metastatic progression of PCa remains an unmet clinical challenge. Resistance to anoikis, a form of cell death initiated by cell detachment from the surrounding extracellular matrix, is one of the cellular processes critical for PCa progression towards aggressive disease. Therefore, further understanding of anoikis regulation in PCa might provide therapeutic opportunities. Here, we discover that PCa tumours with concomitant inhibition of two tumour suppressor phosphatases, PP2A and PTEN, are particularly aggressive, having < 50% 5‐year secondary‐therapy‐free patient survival. Functionally, overexpression of PME‐1, a methylesterase for the catalytic PP2A‐C subunit, inhibits anoikis in *PTEN*‐deficient PCa cells. *In vivo*, PME‐1 inhibition increased apoptosis in *in ovo* PCa tumour xenografts, and attenuated PCa cell survival in zebrafish circulation. Molecularly, PME‐1‐deficient PC3 cells display increased trimethylation at lysines 9 and 27 of histone H3 (H3K9me3 and H3K27me3), a phenotype known to correlate with increased apoptosis sensitivity. In summary, our results demonstrate that PME‐1 supports anoikis resistance in *PTEN*‐deficient PCa cells. Clinically, these results identify PME‐1 as a candidate biomarker for a subset of particularly aggressive *PTEN*‐deficient PCa.

## Graphical abstract

A subset of prostate cancer (PCa) tumours present simultaneous inactivation of two tumour suppressor phosphatases; phosphatase and tensin homolog (PTEN) and protein phosphatase 2A (PP2A). PP2A is inhibited via overexpression of PME‐1. Such cancers are particularly aggressive and often relapse from standard therapy, indicating PME‐1 as a potential clinically applicable biomarker for PCa. Mechanistically, PME‐1 expression protects cancer cells from anoikis, promoting their survival outside the primary tumour.

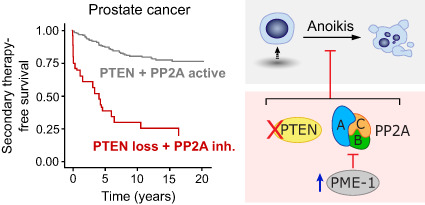

**Abbreviations**
AKT: AKT Serine/Threonine Kinase 1
AR: androgen receptor
BAD: BCL2‐associated agonist of cell death
CAM: chicken embryo chorioallantoic membrane
cGAS‐mCherry: mCherry‐tagged cyclic GMP–AMP synthase
CIP2A: cellular inhibitor of PP2A
cPARP: cleaved poly(ADP‐ribose) polymerase
CRPC: castration‐resistant prostate cancer
DAPI: 4′,6‐diamidino‐2‐phenylindole
ECM: extracellular matrix
EEA1: early endosome antigen 1
ERG: ETS transcription factor ERG
FAK: focal adhesion kinase
GAPDH: glyceraldehyde‐3‐phosphate dehydrogenase
GFP: green fluorescent protein
GFP‐NLS: GFP‐tagged nuclear localization signal
H3K27me3: histone H3 lysine 27 trimethylation
H3K9me3: histone 3 lysine 9 trimethylation
IHC: immunohistochemical
Ki‐67: marker of proliferation Ki‐67
KO: knock‐out
LAP2A/B: lamina‐associated polypeptide 2 alpha/beta
MEF: mouse embryonic fibroblasts
MYC: MYC proto‐oncogene
NL: nuclear lamina
nt: non targeting
NUP98: nucleoporin 98
pAKT: phosphorylated AKT
PARP: poly(ADP‐ribose) polymerase
PCa: prostate cancer
PEG: poly(ethylene glycol)
pFAK: phosphorylated FAK
PLA: proximity ligation assay
PLL‐g‐PEG: poly(l‐lysine)‐g‐poly(ethylene glycol)
PME‐1: protein phosphatase methylesterase 1
pMYC: phosphorylated MYC
PP2A: protein phosphatase 2A
PP2A‐C: catalytic subunit of PP2A
PPP2R2A: PP2A B‐subunit (B55alpha)
PPP2R2C: PP2A B‐subunit (B55gamma)
PTEN: phosphatase and tensin homolog
SET: SET nuclear proto‐oncogene
siCtrl: control siRNA
STS: staurosporine
TMA: tissue microarray
TUNEL: terminal deoxynucleotidyl transferase dUTP nick end labeling

## 1 Introduction

Prostate cancer (PCa) is often detected early and can remain non‐aggressive and non‐metastatic for years [1, 2]. Whereas local PCa is not life‐threatening, transformation from indolent to aggressive disease is clinically the most important transition in PCa progression. Thereby understanding this switch in more detail might provide therapeutic opportunities. Analysis of the evolutionary history of lethal metastatic PCa has revealed that tumour suppressor proteins are particularly lost during transition towards metastatic human PCa [1]. One such tumour suppressor is a dual‐specificity phosphatase Phosphatase and Tensin homolog (PTEN) that is inactivated in a large fraction of high‐grade PCas [3]. Both in human and mice, prostate‐specific *PTEN* deletion leads to an aggressive and metastatic PCa [3, 4, 5]. Protein phosphatase 2A (PP2A) is another tumour suppressor phosphatase that is commonly inactivated in aggressive PCa. Specifically, PP2A B‐subunits PPP2R2A and PPP2R2C (B55α and B55γ) have been reported as either genetically deleted or down‐regulated in human PCa samples [6, 7, 8]. PP2A is inactivated also non‐genetically in human cancers by overexpression of inhibitor proteins such as CIP2A, PME‐1 or SET [9, 10]. CIP2A overexpression clinically associates with castration‐resistant prostate cancer (CRPC) [11], and inhibition of both CIP2A and SET inhibits malignant growth of PCa cells [11, 12]. In contrast, the role of PME‐1 in PCa is currently unknown. PME‐1 inhibits PP2A activity both by directly binding to catalytic centre of the catalytic subunit PP2A‐C, and by regulating the methylation of the C‐terminal leucine 307 on PP2A‐C and thereby inhibiting recruitment of tumour suppressive B‐subunits to the PP2A complex [10, 13, 14]. Thereby, PME‐1 expression levels provide an indirect read‐out for effective PP2A inhibition in cells and in tissues.

One of the hallmarks of PCa progression towards aggressive CRPC is acquisition of resistance towards a specific type of programmed cell death, anoikis [15]. Anoikis is induced by detachment of cells from other cells, or the surrounding extracellular matrix (ECM). Anoikis suppression is not only relevant for indolent PCa cells to acquire anchorage‐independence, but also for survival of PCa cells with metastatic potential in circulation [16, 17]. Thus, understanding the mechanisms driving anoikis resistance of PCa cells could provide novel therapy opportunities for clinical management of PCa by facilitating inhibition of progression to metastatic disease. Anoikis resistance in PCa has been linked to changes in cell adhesion, cytoskeleton, and epigenetics, as well as deregulated intracellular survival pathways [15]. Activated integrin signaling is a central contributor to anchorage‐independence and metastasis [18, 19], and integrin activation correlates with anoikis resistance in PCa cell lines [20, 21]. Recently, epigenetic regulation of histone H3 methylation has also surfaced as an important mechanism regulating apoptosis and anoikis [22, 23, 24]. The role of PP2A in PCa anoikis sensitivity is, however, poorly understood.

In this study, we demonstrate that PP2A inhibitor protein PME‐1, that has not been previously implicated in PCa, has a critical role in anoikis suppression of *PTEN*‐deficient PCa cells both *in vitro* and *in vivo*. Clinically, PCa tumours with inhibition of both tumour suppressor phosphatases *PTEN* (genetic deletion) and PP2A (PME‐1 overexpression) had remarkably short relapse‐free survival introducing PME‐1 as a potential clinically applicable PCa biomarker.

## 2 Material and methods

### 2.1 Cell lines

PC‐3, DU‐145 and LNCaP PCa cell line were obtained from ATCC and cultured in RPMI‐1640. *Pten* ^Δ/Δ^; *Trp53* ^Δ/Δ^; lsl‐tdTomato mouse embryonic fibroblasts were provided by Lloyd Trotman and cultured in DMEM. All growth media were supplemented with 10% heat‐inactivated FBS (Biowest, Nuaillé, France), 2 mmol·L^−1^ L‐glutamine, penicillin (50 units·mL^−1^) and streptomycin (50 μg·mL^−1^). Cells were regularly tested and confirmed to be mycoplasma‐negative and were grown at 37 °C in a humidified atmosphere of 5% CO_2_.

### 2.2 siRNAs

Control (Ctrl): CGU ACG CGG AAU ACU UCG A

PME‐1 (#1): GGA AGU GAG UCU AUA AGC A

PME‐1 (#2): GAA UGA AAC UGG CAA GGA U

PME‐1 (#3): GCU AUU GAA UGG AGU GUG A

PME‐1 (#4): UCA UAG AGG AAG AAG AAG A

PME‐1 (#5): AGG UCA AGA AUC CUG AAG A

All siRNAs were ordered from Eurofins Genomics (MWG).

### 2.3 Antibodies

The following antibodies were used at the indicated dilutions: PME‐1, Santa Cruz Biotechnology (Dallas, TX, USA) sc‐20086 (H‐226), Western blotting 1 : 1000; PME‐1, Santa Cruz Biotechnology sc‐25278 (B‐12), Immunohistochemistry 1 : 1000, Immunofluorescence 1 : 100; cleaved PARP‐1, Abcam (Cambridge, UK) ab32064 [E51], Western blotting 1 : 1000; GAPDH, HyTest 5G4‐6C5, Western blotting 1 : 5000; c‐MYC, Abcam ab32072 [Y69], Western blotting 1 : 1000; p‐Myc S62, Abcam ab78318, Western blotting 1 : 1000; AKT1/2/3, Cell Signaling Technology #9272, Western blotting 1 : 2000; p‐AKT S473, Cell Signaling Technology #4060, Western blotting 1 : 1000; Lamin‐A/C, Santa Cruz Biotechnology sc‐7292 (636), Immunofluorescence 1 : 250; Lamin‐A/C, Santa Cruz Biotechnology sc‐6215 (N‐18), Western blotting 1 : 1000; p‐Lamin‐A/C S392, Abcam ab58528, Western blotting 1 : 5000; EEA1, Santa Cruz Biotechnology sc‐137130 [G4], Immunofluorescence 1 : 100; p‐FAK Y397, Cell Signaling Technology #8556, Immunofluorescence 1 : 100; Histone H3K9me3, Cell Signaling Technology #13969 [D4W1U], Immunofluorescence 1 : 500; Histone H3K27me3, Cell Signaling Technology #9733 [C36B11], Immunofluorescence 1 : 500; PPP2R2A, Cell Signaling Technology #5689, Western blotting 1 : 1000.

### 2.4 siRNA/plasmid transfection

For siRNA transfections, Lipofectamine RNAiMAX (Thermo Fisher Scientific, Waltham, MA, USA) was used following the manufacturer's instructions. 2.5 × 10^5^ cells were seeded on a 6‐well plate 1 day before transfection to reach 60–70% confluency. Cells were then transfected with 25 pmol siRNA and 7.5 μL RNAiMAX per well and assayed 48–72 h after transfection, unless otherwise stated. For plasmid transfections, Lipofectamine 3000 (Thermo Fisher Scientific) was used following the manufacturer's instructions. 3–4 × 10^5^ cells were seeded on a 6‐well plate 1 day before transfection to reach 80–90% confluency. Cells were then transfected with 2.5 μg DNA, 5 μL P3000 reagent and 3.75 μL Lipofectamine 3000 per well and assayed 24–48 h after transfection, unless otherwise stated.

### 2.5 Colony formation assay

Cells were reseeded at low confluency (1–4 × 10^3^) on a 12‐well plate 24 h after siRNA transfection and grown for around 10 days to allow the formation of cell colonies. Colonies were then fixed with ice‐cold methanol and stained with 0.2% crystal violet solution (in 10% ethanol) for 10 min at room temperature. Excess stain was removed by repeated washing with PBS. Plates were dried and scanned with Epson perfection V700 scanner. Quantifications were performed with ColonyArea ImageJ plugin [25], and graphs were plotted using the area % values.

### 2.6 Anchorage‐independent colony formation assay

For the anchorage‐independent colony formation assay, which typically correlates with *in vivo* tumourigenicity, 2 × 10^4^ cells were resuspended in 1.5 mL of growth medium containing 0.4% agarose (4% Agarose Gel, Gibco/Thermo Fisher Scientific; top layer) and plated on 1 mL bottom layer containing growth medium and 1.2% agarose in a 12‐well plate. After 14 days of growth, colonies were stained overnight with 1 mg·mL^−1^ nitro blue tetrazolium chloride (NBT; Molecular Probes, Eugene, OR, USA) in PBS. Colonies were imaged using a Zeiss SteREO Lumar V12 stereomicroscope (Zeiss, Oberkochen, Germany). Analysis was done using the imagej software. First, the background was subtracted using the rolling ball function with a radius of 50 μm, then auto‐thresholding was applied to separate the colonies. Area percentage was calculated using the imagej built‐in function ‘Analyze Particles’. Particles smaller than 500 μm^2^ were not considered colonies, and were excluded from the analysis.

### 2.7 Western blotting

Western blot protein lysates were prepared in 1× RIPA buffer (150 mm sodium chloride, 1.0% NP‐40, 0.5% sodium deoxycholate, 0.1% SDS and 50 mm Tris, pH 7.5) containing PhosSTOP™ phosphatase inhibitors and cOmplete™ EDTA‐free protease inhibitors (Roche). DNA in the protein samples was sheared by sonication and the amount of protein was estimated using Pierce™ BCA Protein Assay Kit (Thermo Fisher Scientific). Lysates were separated on 4–20% Mini‐PROTEAN® TGX™ Gels (Bio‐Rad, Hercules, CA, USA) and transferred by wet blotting to PVDF membranes (Merck Millipore, Burlington, MA, USA), or via semi‐dry transfer to nitrocellulose membranes (Bio‐Rad). Unspecific antibody binding was blocked with 5% non‐fat dry milk in TBST. Incubation for the primary antibodies was performed overnight at 4 °C in either 5% non‐fat dry milk, or in 5% BSA for the phospho‐specific antibodies. For detection, HRP‐labeled secondary antibodies (DAKO/Agilent, Santa Clara, CA, USA) followed by incubation with Pierce™ ECL Western Blotting Substrate (Thermo Fisher Scientific) were used. Alternatively, LI‐COR Biosciences secondary antibodies (IRDye 680 or IRDye 800) were used, followed by detection by Odyssey® Imaging Systems or Bio‐Rad Laboratories ChemiDoc Imaging Systems.

### 2.8 Generation of *Pten* ^Δ^ ^/Δ^; Trp53^Δ^ ^/Δ^ mouse embryonic fibroblasts

*Pten* ^Δ/Δ^; *Trp53* ^Δ/Δ^; lsl‐tdTomato MEFs were generated as previously described [26]. To stably overexpress PME‐1 in these cells, PME‐1 cDNA was cloned into pCSCIW‐2 lentiviral construct which was then packaged into lentiviral particles. Following transduction, GFP positive cells were sorted and overexpression of PME‐1 was confirmed by western blotting.

### 2.9 CRISPR/Cas9‐mediated PME‐1 knock‐out

To generate PME‐1 knock‐out cells, PC‐3 cells were transduced with lentivirus containing lentiCas9‐Blast construct (Addgene, Watertown, MA, USA; #52962) and selected with growth medium containing 4 μg·mL^−1^ blasticidin for approximately 1 week. Afterwards, the cells were infected with lentivirus sgRNA against PME‐1 exon 3 (sequence: ACTTTTCGAGTCTACAAGAGTGG) cloned into pKLV‐flipedU6gRNA_PB_BbsI_PGKpuro2ABFP construct. After puromycin selection (2 μg·mL^−1^ in growth medium), PME‐1 KO on the protein level was confirmed by western blotting. Single cell clones were obtained by cell sorting and the knock‐out was confirmed by sequencing (Primers. Forward CACCGCTTTTCGAGTCTACAAGAGGT; reverse TAAAACGAAGATCTGTCTGCAGAAAC). Cas9‐ and non‐targeting gRNA‐expressing cells served as a negative control in the functional assays.

### 2.10 Chick chorioallantoic membrane assay

The chorioallantoic membrane (CAM), one of three extraembryonic membranes formed during chick development, provides a highly vascularized and immunodeficient test environment, which is frequently utilized to address cancer‐relevant questions. Fertilized chicken eggs were purchased from a certified hatchery (LSK Poultry Oy, Laitila, Finland). According to the Directive 2010/63/EU of the European Parliament on the protection of animals used for scientific purposes, the CAM model system does not raise any ethical or legal concerns. To start chick embryonic development for the CAM assay, fertilized eggs were kept rotating in an incubator at 37 °C and 50–60% humidity for 4 days. After the initial incubation, a small hole was introduced at the sharp edge of the egg and sealed with parafilm. Following four more days of incubation on stationary racks, the hole was enlarged and a plastic ring was placed on top of blood vessels in the CAM. Next, 1 × 10^6^ PC‐3 cells in 20 μL volume of a 1 : 1 mixture of ice‐cold PBS and matrigel were pipetted inside the ring on the membrane. The hole was then covered with parafilm, and the eggs were incubated for three more days. At day 12 of embryonic development, the animals were sacrificed by freezing the eggs for 15 min, and the tumour cell mass was dissected from the membrane and processed for further analysis.

### 2.11 Immunohistochemistry

PCa tissue microarrays have been described previously [27]. Hematoxylin/eosin staining and immunohistochemistry were performed on 3‐μm‐thick sections of 4% paraformaldehyde‐fixed and paraffin‐embedded tissues. Following rehydration, endogenous peroxidase was blocked by incubation in 50% MetOH, 1% H_2_O_2_. Subsequent antigen retrieval was performed with the 2100 Retriever (Aptum Biologics, Southampton, UK) in R‐Universal Buffer. Unspecific antibody binding was blocked with 10% goat serum in 2% BSA/PBS prior to overnight incubation at 4 °C with the primary antibody. For detection, the DAKO EnVision peroxidase system, followed by incubation with 0.01% diaminobenzidine (Sigma‐Aldrich, St. Louis, MO, USA) was used.

### 2.12 Zebrafish *in vivo* dissemination assay

The zebrafish used in experiments were bred in the Zebrafish Core of Turku Bioscience Centre. The zebrafish strain was originally a kind gift of Dr. Adam Hurlstone (University of Manchester). The xenotransplantation of zebrafish embryos was performed as described in detail in [28] with some modifications. PC‐3 cells were washed with PBS, stained with CellTracker Green CMFDA dye (5 μm, Thermo Fisher Scientific) and detacher and detached with trypsin–EDTA in a single incubation step at 37 °C. Subsequently, cells were pelleted by centrifugation and washed with PBS twice. This was followed by filtration through 40 μm mesh into Falcon FACS tube (Corning, Corning, NY, USA, 352235) and pelleting cells by centrifugation. Finally, cells were resuspended into 30 μL of injection buffer (2% PVP in PBS) and kept on ice until transplanted. Zebrafish (*Danio rerio*) of pigmentless casper strain (*roy* ^−/−^; *mitfa* ^−/−^) [29] was used in the experiments under license no. MMM/465/712–93 (issued by the Finnish Ministry of Forestry and Agriculture) and following legislation: the European Convention for the Protection of Vertebrate Animals used for Experimental and other Scientific Purposes and the Statutes 1076/85 and 62/2006 of The Animal Protection Law in Finland and EU Directive 86/609. The embryos were obtained through natural spawning, and were cultured in E3‐medium (5 mm NaCl, 0.17 mm KCl, 0.33 mm CaCl_2_, 0.33 mm MgSO_4_) at 33 °C. At 2 or 3 days post‐fertilization, the embryos were anesthesized with 200 mg·mL^−1^ Tricaine and embedded in 0.7% low‐melting point agarose. Subsequently, the cell suspension was microinjected into common cardinal vein (duct of Cuvier) of the embryo using glass capillaries (Transfertip), CellTramVario microinjector and InjectMan micromanipulator (all from Eppendorf, Hamburg, Germany). Embryos were liberated from the agarose gel using forceps and successfully transplanted embryos were selected to the experiment. After overnight incubation at 33 °C, the embryos were anesthesized again with Tricaine and imaged using Zeiss StereoLumar V12 fluorescence stereomicroscope. The number of surviving cells was counted manually from the images using imagej.

### 2.13 Proximity ligation assay

Cells were grown to approximately 80% confluency on sterilized coverslips, fixed for 10 min in 4% PFA and permeabilized for 10 min with 0.5% Triton X‐100 in TBS. The subsequent steps were completed following the manufacturer's instructions (Sigma). Briefly, unspecific antibody binding was blocked with the provided blocking solution for 30 min. The slides were then incubated with the primary antibodies overnight at 4 °C, with the mouse and rabbit probes for 1 h at 37 °C, with the ligation mix for 30 min at 37 °C and the amplification mix for 100 min at 37 °C. The washing steps in‐between the individual steps were carried out with Buffer A (Sigma) and the final washing step with Buffer B (Sigma). Slides were mounted in Mowiol mounting medium and imaged with a Zeiss LSM780 confocal microscope.

### 2.14 Investigation of nuclear envelope integrity

PC‐3 cells were transfected with PME‐1 or control siRNA as indicated above. After 24 h, the same cells were transfected with both pCDH‐CMV‐NLS‐copGFP‐EF1‐blastiS and pCDH‐CMV‐cGASE225A/D227A‐mCherry2‐EF1‐blastiS [30] in a 1 : 1 ratio and incubated overnight. Finally, the cells were detached, transferred onto 0.5 kPa hydrogels and grown for an additional 24 h before fixing. Lamin‐A,C, NLS‐GFP, cGAS‐mCherry and DNA were visualized using (immune)fluorescence microscopy. After thresholding, individual cells were stratified and quantified based on the presence/absence of cytoplasmic NLS and cGAS foci.

### 2.15 Cell‐matrix interaction assay

To quantitatively control the degree of cell‐matrix interaction, 1 mg·mL^−1^ poly‐L‐lysine‐grafted polyethylene glycol (PLL(20)‐g[3.5]‐PEG(2), from SuSoS AG) and 1 mg·mL^−1^ 50% biotinylated PLL‐g‐PEG (PLL(20)‐g[3.5]‐PEG(2)/PEG(3.4)‐biotin(50%), from SuSoS AG) stock solutions were mixed with 10 mm Hepes (pH 7.4) to create 0.1 mg·mL^−1^ working solutions with 0%, 0.1%, 0.25%, 1%, 5% or 20% biotinylated PLL‐g‐PEG. A plastic 96‐well plate was coated with each of the different stock solutions for 1 h at room temperature and washed twice with PBS. Next, the coated wells were immersed in approximately 250 ng·cm^−2^ of streptavidin‐conjugated (FastLink Streptavidin Labeling Kit, Abnova, Taipei, Taiwan) fibronectin fragment, FNIII(7–10), in PBS and incubated for 1 h at room temperature. Afterwards, the wells were washed three times with PBS. To evaluate the degree of cell adhesion to the coated wells, PC‐3 cells were detached using HyQTase (HyClone Laboratories, Logan, UT, USA, SV30030.01) and 1 × 10^4^ cells were seeded into each well. The cells were left to adhere and spread for 30 min, after which the wells were washed twice with PBS while avoiding draining them completely. The cells were fixed with 4% PFA/PBS for 5 min, washed three times with PBS and visualized by incubating the wells in 0.2% (w/v) crystal violet (Sigma)/10% ethanol for 15 min at room temperature. The wells were washed three times with PBS, air dried, and 100 μL of 10% acetic acid per well was used to resolubilize the stain. The results (A595) were read using a spectrophotometer (Multiskan Ascent, Thermo Fisher Scientific).

To investigate the degree of anoikis in PC‐3 cells grown on surfaces with varying amounts of integrin ligand, cells that had been transfected with control or PME‐1‐targeting siRNAs approximately 48 h prior to the experiment were seeded on a new 96‐well plate coated with 0–20% biotinylated PLL‐g‐PEG and streptavidin‐FNIII(7–10), at 5 × 10^3^ cells per well. Each well was supplemented with 1 : 200 Annexin V‐FITC (eBioscience), and additional wells with 20% biotinylated PLL‐g‐PEG and FNIII(7–10) were supplemented with 1 μm staurosporine (STS) (Merck Millipore) to serve as a positive control for apoptosis. The plate was imaged every hour for > 2.5 days and analyzed for the presence of Annexin V‐positive cells and debris using IncuCyte S3 (Essen Bioscience, Welwyn Garden City, UK).

### 2.16 Actin cytoskeleton disruption

Plastic 6‐well plates and 8‐well microscopy slides (Ibidi μ‐Slide) were coated overnight at 4 °C with 2.5 μg·mL^−1^ of bovine plasma fibronectin (Merck‐Millipore, 341631) and collagen type I (Sigma, C8919). On the following day, control and PME‐1 KO PC‐3 cells were seeded on the 6‐well plates and microscopy slides at approximately 30% seeding density and supplemented with DMSO (control) or 0.1–10 μm of Cytochalasin D (Sigma, C8273‐1MG) 3 h later. Cells on the microscopy slides were fixed after two‐hour treatment and processed into fluorescence microscopy samples as indicated below. The remaining cells were lysed after 24 h and analyzed for the presence of cleaved PARP by western blotting.

### 2.17 Immunofluorescence

Cells were fixed with 4% PFA for 10 min at room temperature, and simultaneously permeabilized and blocked with 0.3% Triton in 10% horse serum (Gibco) for 15 min at room temperature. All samples were incubated in primary antibodies (diluted in 10% horse serum) overnight at 4 °C, and stained with secondary antibodies for 1–2 h at room temperature on the following day. Appropriate Alexa Fluor‐conjugated secondary antibodies (Thermo Fisher Scientific) were used at a 1 : 300 dilution in PBS. Nuclei were counterstained with DAPI (4′,6‐diamidino‐2‐phenylindole) and filamentous actin with Alexa Fluor 488‐conjugated phalloidin (Thermo Fisher Scientific).

### 2.18 Polyacrylamide hydrogels

Thirty‐five‐millimeter glass bottom dishes (MatTek Corporation, Ashland, MA, USA, P35G‐1.0‐14‐C) were treated with 100 μL of Bind‐silane solution [7.14% Bind‐silane (Sigma, M6514) and 7.14% acetic acid in absolute ethanol] for 15 min, washed twice with absolute ethanol and left to dry completely. A pre‐polymer mix comprising 5.4% acrylamide (Sigma) and 0.043% N,N′‐methylenebisacrylamide (Sigma) in PBS was prepared to obtain hydrogels with an elastic modulus of appoximately 0.5 kPa [31]. Polymerization was initiated by adding 2.5 μL of 20% ammonium persulfate (Bio‐Rad) and 1 μL of N,N,N′,N′‐tetramethylethylenediamine (Sigma). The solution was vortexed, 13 μL was added on top of the glass bottom dish, a 13 mm glass coverslip was placed on the drop and the gel was left to polymerize for 1 h at room temperature. After polymerization, the dish was filled with PBS and the coverslip was carefully removed. Hydrogels were made permissive for protein binding by incubating them in 500 μL of Sulfo‐SANPAH solution [0.2 mg·mL^−1^ Sulfo‐SANPAH (Sigma, 803332) and 2 mg·mL^−1^ N‐(3‐dimethylaminopropyl)‐N′‐ethylcarbodiimide hydrochloride (Sigma, 03450) in 50 mm HEPES] for 30 min on slow agitation, followed by a 10 min UV exposure (~ 30 mW·cm^−2^, 253.7 nm). Activated gels were washed three times with PBS to get rid of residual Sulfo‐SANPAH. Alternatively, pre‐activated polyacrylamide hydrogels of variable stiffness were ordered from Matrigen Life Technologies. Thirty‐five‐millimeter glass bottom dishes (SV3510‐EC‐0.5) were used for imaging and 6‐well plates (SW6‐EC‐0.5, SW6‐EC‐50) for growing cells for lysis. All hydrogels were functionalized with bovine plasma fibronectin and collagen type I by incubating the dishes in 5 μg·mL^−1^ of each protein for 1–2 h at 37 °C, or overnight at 4 °C, before use. For western blot analysis of cells on hydrogels, 0.5 and 50 kPa hydrogel‐coated 6‐well plates were immersed in cell culture medium for 30 min and seeded with 300000 PME‐1 or control siRNA‐treated PC‐3 cells per well. The cells were grown on the gels for 24 h. Once the cultures reached 80–90% confluency, the cells were washed once and scraped into cold PBS, spun down and lysed in RIPA buffer as described above. For immunofluorescence experiments, PC‐3 cells were seeded on 0.5 kPa hydrogel‐coated glass bottom dishes and grown for an additional 24 h before fixing and sample preparation.

### 2.19 Microscopy and image analysis

Fluorescent specimens were imaged using a spinning disk or laser scanning confocal microscope. The spinning disk system used was a Marianas spinning disk confocal microscope with a Yokogawa CSU‐W1 scanning unit, controlled by slidebook 6 software (Intelligent Imaging Innovations, Denver, CO, USA). The objective used was a 40×/1.1 W LD C‐Apochromat objective (Zeiss), and images were acquired using an Orca Flash4 sCMOS camera (Hamamatsu Photonics, Hamamatsu, Japan). The laser scanning confocal microscope used was an LSM780, controlled by Zen 2010 (Zeiss), and the objective used was a 40×/1.2 W C‐Apochromat objective (Zeiss). Images were analyzed using imagej (National Institutes of Health) and cellprofiler (Broad Institute) software.

### 2.20 Statistical analysis

Statistical analyses and plotting were performed using graphpad prism v6.05 (GraphPad Software, San Diego, CA, USA). The names and/or numbers of individual statistical tests, samples and data points are indicated in figure legends. Unless otherwise noted, all results are representative of a minimum of two independent experiments and two‐tailed *P*‐values are reported.

## 3 Results

### 3.1 PME‐1 overexpression in prostate cancer associates with *PTEN* loss and therapy relapse

**Fig. 1:**
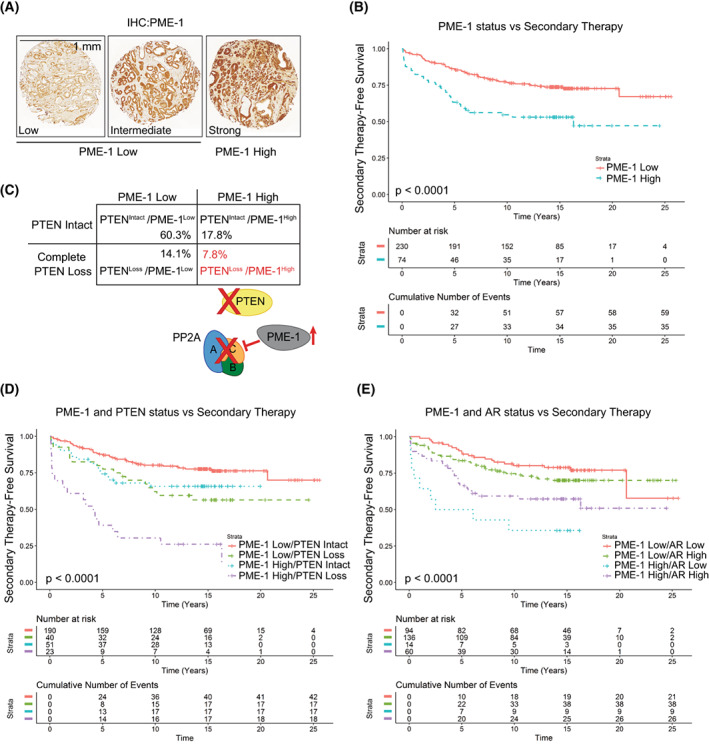
PME‐1 overexpression associates with *PTEN* loss and therapy relapse of *PTEN‐*negative prostate cancer patients. (A) PME‐1 protein expression in PCa tissue microarray material from 358 patients treated primarily with radical prostatectomy in the Helsinki University Hospital between 1983 and 1998 was assessed by immunohistochemical stainings. PME‐1 protein content was scored using 4‐tier scale; negative (not shown), low, intermediate and strong expression. In subsequent clinical analysis negative‐intermediate immunopositivity of PME‐1 was considered as PME‐1 Low, whereas strong PME‐1 immunopositivity was considered as PME‐1 High. Scale bar 1 mm. (B) Kaplan‐Meyer analysis of time to secondary therapies after primary treatment based on PME‐1 status alone. *P*‐value by log rank test. (C) Assessment of the status of PME‐1 and *PTEN* in the patient populations and the putative effect on PP2A activity. (D, E) Kaplan‐Meyer analysis of time to secondary therapies after primary treatment based on PME‐1 status in combination with *PTEN* deletion (D) and AR expression (E). *P*‐values by log rank test.

PME‐1 protein expression and its clinicopathological associations were evaluated in PCa tissue microarray (TMA) material comprising 358 patients treated primarily with radical prostatectomy in the Helsinki University Hospital between 1983 and 1998. The clinical cohort has previously been described in detail [5, 27] (see Table S1 for demographics). The specificity of the PME‐1 antibody for immunohistochemical (IHC) staining has been validated previously [32]. PME‐1 expression was scored using a four‐tier scale of negative, low, intermediate, and strong expression. Each patient had one benign core and three cancerous cores in the TMA. PME‐1 expression was lower in benign prostate compared with cancerous prostate in most patients (Table S2). In the survival analysis, the maximum values of cancer‐related PME‐1 scores out of three cancerous cores in the TMA were used and each patient was dichotomized as either low or high (negative, low, intermediate vs. strong staining; Fig. 1A). In the correlation analysis with clinical variables, strong PME‐1 protein expression correlated with higher grade group and advanced stage (Table 1). We also correlated PME‐1 status with previously assessed *PTEN*, ERG and AR status [5, 27]. Notably, high PME‐1 expression significantly associated not only with complete *PTEN* loss but also with ERG positivity and high AR expression status (Fig. S1A).

**Table 1:** Univariate analysis of PME IHC and clinical variables.

	PME IHC low (*n* = 238)	PME IHC high (*n* = 82)	*P*‐value^a^
Age at RP, years (mean, SD) (*n* = 320)			
< 60	81 (34.0)	24 (29.3)	0.515
60–70	132 (55.5)	46 (56.1)	
> 70	25 (10.5)	12 (14.6)	
Preoperative PSA, ng·mL^−1^ (*n*, %) (*n* = 254)			
≤ 10.0	100 (52.1)	32 (51.6)	0.995
10.1–20.0	59 (30.7)	19 (30.6)	
> 20.0	33 (17.2)	11 (17.8)	
Grade group at RP (*n*, %) (*n* = 320)			
1	74 (31.1)	5 (6.1)	**< 0.001**
2	65 (27.3)	17 (20.7)	
3	73 (30.7)	36 (43.9)	
4	22 (9.2)	17 (20.7)	
5	4 (1.7)	7 (8.5)	
pT (*n*, %) (*n* = 299)			
2	150 (67.3)	28 (36.8)	**< 0.001**
3–4	73 (32.7)	48 (63.2)	
Lymph node status (*n*, %) (*n* = 314)			
Negative	228 (97.4)	78 (97.5)	1.000
Positive	6 (2.6)	2 (2.5)	
Secondary therapy (*n*, %) (n = 320)			
No	171 (71.8)	39 (47.6)	**< 0.001**
Yes	67 (28.2)	43 (52.4)	
Death from any cause (*n*, %) (*n* = 320)			
Yes	104 (43.7)	46 (56.1)	0.055
No	134 (56.3)	36 (43.9)	
Death from prostate cancer (*n*, %) (*n* = 320)			
Death due to PC	20 (8.4)	11 (13.4)	0.197
Alive, or dead from other causes	218 (91.6)	71 (86.6)	

^a^ Pearson's chi‐square.

Patients with high tumour PME‐1 expression had significantly shorter time to secondary therapies after primary treatment (i.e., relapse‐free survival; Fig. 1B), indicative of clinical relevance of PME‐1 in human PCa. Linked to the association between *PTEN* loss and high PME‐1 expression (Fig. S1A), we asked whether patients with tumours defective for both tumour suppressor phosphatases' functions (*PTEN* and PP2A) would present with a particularly aggressive disease. Patients with *PTEN* ^loss^/PME‐1^high^ tumour phenotype constituted a sub‐cohort of approximately 8% of patients (Fig. 1C). *PTEN* ^loss^/PME‐1^high^ status correlated with remarkably aggressive tumours, with about 40% 5‐year secondary‐therapy free survival among the cohort (Fig. 1D). Similarly, albeit less prominent co‐operative effects were observed with PME‐1 overexpression and either AR (Fig. 1E) or ERG expression (Fig. S1B).

These results reveal a clinical relevance of PME‐1 in PCa. Notably, they identify a very aggressive PCa subpopulation associated with concomitant inhibition of PTEN and PP2A tumour suppressor phosphatases.

### 3.2 PME‐1 promotes anchorage‐independent growth of prostate cancer cells

**Fig. 2:**
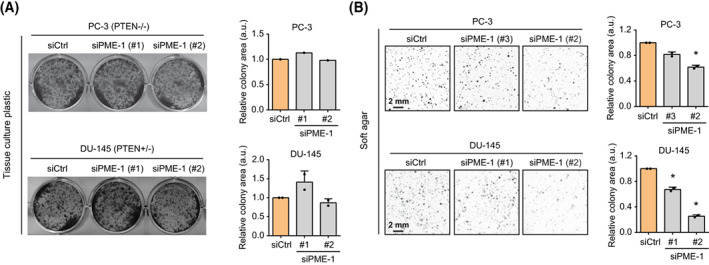
PME‐1 promotes anchorage‐independent growth of prostate cancer cells. (A) The effect of PME‐1 depletion, using two independent siRNAs, was investigated by colony formation assays (10 days of growth) in two *PTEN*‐deficient PCa cells lines PC‐3 and DU‐145. (Left) Representative images of the wells. (Right) Bar graphs depicting the quantified data, mean ± SD of one (PC‐3) or two (DU‐145) independent experiments. (B) The effect of PME‐1 knock‐down on anchorage‐independent growth in soft agar assays (14 days of growth) in both PC‐3 and DU‐145 cells. (Left) Representative images depicting the colonies. (Right) Bar graphs displaying the quantified data, mean ± SD of two independent experiments. *P < 0.05, Welch's *t*‐test.

Acknowledging the clinical indications for a link between PME‐1 and PTEN, we tested the impact of PME‐1 depletion on colony growth of two *PTEN* ‐deficient PCa cell lines, PC‐3 (*PTEN* null) and DU‐145 (*PTEN* heterozygous). PME‐1 silencing by two independent siRNAs did not affect colony growth of either of the cell lines in 2D adherent cell culture conditions (Fig. 2A). However, PME‐1 silencing decreased anchorage‐independent growth of both cell lines in soft agar (Fig. 2B). The context‐dependent role of PME‐1 in supporting anchorage‐independent growth, but being redundant for cell growth in 2D adherent conditions is fully consistent with the established requirement of PP2A inhibition for anchorage‐independence of transformed human cells [33].

**Fig. 3:**
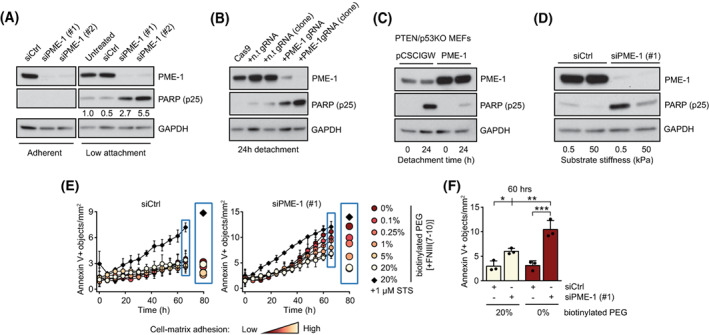
PME‐1 promotes anoikis resistance in *PTEN*‐deficient prostate cancer cells. (A) siCtrl‐ and siPME‐1‐transfected PC‐3 cells were plated 72 h post‐transfection on normal cell culture or low‐attachment plates for 24 h, before collection and lysis, and subsequently analyzed by western blotting for cleaved PARP‐1 (cPARP (p25)). Shown is a representative result from six independent experiments. (B) Apoptosis induction, as measured by PARP cleavage, in CRISPR/Cas9 generated PC‐3 cells, with and without non‐targeting gRNA (lanes 1–3), a pool of PME‐1 gRNA transfected cells (lane 4), and a single cell subclone of PME‐1 targeted cells (lane 5), after 24 h detachment. Shown is a representative result from three independent experiments. (C) The effect of stable PME‐1 overexpression in mouse embryonic fibroblasts (MEFs) from a *PTEN/p53*KO mouse model, after 24 h of detachment, was assessed by western blotting for cleaved PARP. Shown is a representative result from two independent experiments. (D) Mechanosensitive PARP cleavage in PME‐1‐depleted PC‐3 cells. The cells were cultured on soft (0.5 kPa) or stiff (50 kPa) polyacrylamide hydrogel for 24 h before being scraped into PBS, spun down, lyzed and analyzed for protein expression. Shown is a representative result from four independent experiments. (E) The number of Annexin V positive objects, a marker for apoptosis, in cultures of siCtrl‐ and siPME‐1‐transfected PC‐3 cells upon varying degrees of cell‐matrix interaction. The cells were grown on PEG (Poly(ethylene glycol))‐coated surfaces with 0–20% biotinylated compound bound to integrin ligand, fibronectin fragment FNIII(7–10). STS = staurosporine. Mean ± SD of three independent experiments. (F) Annexin V positive objects in PC‐3 cultures with no or high levels of integrin ligand, after 60 h of incubation. Mean ± SD of three independent experiments. *P < 0.05, **P < 0.01, ***P < 0.001, one‐way ANOVA with Bonferroni's multiple‐comparison test.

To further assess whether PME‐1 is particularly relevant for survival under anchorage‐independent conditions, we evaluated apoptosis induction in control and PME‐1 siRNA‐transfected PC‐3 cells cultured either on tissue culture plastic or low attachment plates. We observed a strong synergy between PME‐1 inhibition (by two independent siRNA oligos) and low attachment culture conditions in apoptosis induction measured by PARP cleavage (Fig. 3A). Similar PME‐1 depletion‐induced apoptosis in low‐attachment conditions was observed in both DU‐145 cells and LNCaP, a *PTEN* null, AR high PCa cell line (Fig. S1C,D). To rule out the possibility of siRNA off‐target effects, we created PC‐3 PME‐1 knock‐out (KO) cells (pool or single‐cell clone) by CRISPR/Cas9. These cells displayed dose‐dependent apoptosis induction on PME‐1 loss and following detachment from substrate (Fig. 3B). The functional co‐operation of PTEN and PP2A was interrogated further in a genetically defined model system: PME‐1 overexpression in *PTEN*/*p53* KO mouse embryonic fibroblasts (MEFs) [26]. In accordance with siRNA and CRISPR/Cas9 results, overexpression of PME‐1 in *PTEN/p5*3 KO MEFs prevented PARP cleavage upon 24 h incubation in low‐attachment conditions (Fig. 3C). The relative baseline PME‐1 protein expression levels across different cell models are shown in Fig. S1E. Taken together, these data indicate an important role of PME‐1 in supporting anchorage‐independent growth across *PTEN*‐deficient cells.

In addition to low‐attachment conditions, culturing cells on low‐stiffness matrix mimics a situation where cells are poorly able to transmit adhesion‐dependent signals from their surroundings (i.e. anchorage‐independent growth). Previously, it was shown that non‐transformed cells succumb to anoikis‐type cell death when plated on soft substrates, whereas cancer cells do not die under the same conditions [34, 35]. To test whether PME‐1 expression contributes to apoptosis resistance of cancer cells on soft matrix, control and PME‐1 siRNA transfected PC‐3 cells were plated on low (0.5 kPa) or high (50 kPa) stiffness hydrogels functionalized with ECM components (fibronectin and collagen I). Consistent with published results from other cancer cells [34], control PC‐3 cells showed only a very small increase in PARP cleavage on soft substrate after 24 h. However, PME‐1 depletion sensitized the cells to apoptosis induction selectively in low‐stiffness conditions (Fig. 3D). Integrin‐mediated cell adhesion to ECM ligands activates outside‐in signaling pathways to promote cell proliferation and viability [18, 20]. To test if the apoptosis of PME‐1‐depleted cells scales with the degree of adhesion signaling, we exposed siCtrl‐ and siPME‐1 transfected cells to surfaces functionalized with increasing concentrations of integrin ligand (fibronectin‐fragment FNIII(7–10)), while non‐specific interactions with the plastic surface were prevented using an anti‐fouling agent (Fig. S2A). Consequently, cell adhesion to the surface was fully dependent on the concentration of the integrin ligand (Fig. S2B). Apoptosis (Annexin V signal) was highest in PME‐1‐silenced cells with no available integrin ligand (0%) and decreased proportionally with increasing integrin‐mediated adhesion, whereas control cells were insensitive to lack of adhesion and showed marked apoptosis only upon STS treatment, which was included as a positive control (Fig. 3E,F and Fig. S2C).

In summary, these results demonstrate the potential of PME‐1 overexpression in protecting *PTEN*‐deficient PCa cells from anoikis.

### 3.3 PME‐1 supports *in vivo* anoikis resistance and survival of prostate cancer cells in circulation

**Fig. 4:**
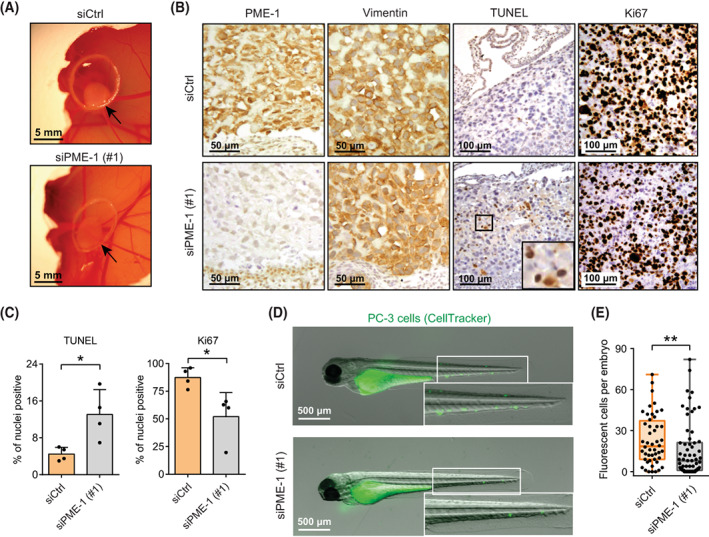
PME‐1 supports *in vivo* anoikis resistance and survival of prostate cancer cells in circulation. (A) The effect of PME‐1 on anchorage‐independent growth of PC‐3 cells on chick chorioallantoic membrane (CAM). PC‐3 cells were transiently transfected with either control siRNA (siCtrl) or PME‐1‐targeting siRNA (siPME‐1), and 24 h post‐transfection placed on the CAM. Growth of tumours was followed for 3–5 days. Shown are representative examples from three replicate experiments. (B) Immunohistological staining of dissected tumours using antibodies for PME‐1, Vimentin, TUNEL and Ki67. Shown are representative images from three (Ki67) or two (TUNEL) replicate experiments. Scale bar 50 μm for PME‐1 and Vimentin stainings and 100 μm for TUNEL and Ki67. (C) Quantification of TUNEL‐ and Ki‐67‐positive nuclei in the excised CAM tumours. Mean ± SD from a representative experiment with four eggs per condition, *P < 0.05, Mann–Whitney test. (D) The survival of siCtrl‐ and siPME‐1‐transfected PC‐3 cells in circulation. The cells were microinjected into the common cardinal veins of zebrafish embryos 72 h post‐transfection. After overnight incubation the embryos were imaged by fluorescence stereomicroscopy. Scale bar 500 μm. (E) The number of surviving fluorescent tumour cells per embryo from (D). Box plots depicting the range, 25th, 50th and 75th percentiles of the data overlaid with the individual data points combined from three independent experiments. **P < 0.01, Mann–Whitney test.

To test the *in vivo* relevance of PME‐1‐mediated inhibition of PCa cell anoikis, we grew either siCtrl‐ or siPME‐1‐transfected PC‐3 xenografts on chicken embryo CAMs. Tumours formed by PME‐1‐depleted cells were overall more translucent, suggesting decreased tumour growth (Fig. 4A). In accordance with the anoikis suppressing activity of PME‐1, histological analyses of the dissected tumours revealed increased apoptosis (TUNEL positivity) in tumours derived from siPME‐1‐transfected cells (Fig. 4B,C). Tumour suppression was further confirmed by the reduced number of Ki‐67 positive cells in tumours derived from PME‐1‐depleted PC‐3 cells (Fig. 4B,C).

Anoikis resistance is crucial for tumour cell dissemination through circulation. To test whether high PME‐1 expression could also suppress PCa cell death in circulation *in vivo*, we examined the survival of control and PME‐1‐depleted, fluorescent PC‐3 cells microinjected into the common cardinal vein of zebrafish embryos [28]. Stereomicroscope imaging and quantification indicated that control cells were detected more frequently in the vasculature after 24 h compared with the PME‐1‐silenced cells (Fig. 4D,E), demonstrating that PME‐1 supported survival of circulating PC‐3 cells. These results are indicative of PME‐1‐mediated anoikis resistance of PCa cells *in vivo*.

### 3.4 The potential mechanisms behind PME‐1‐mediated anoikis resistance

A recent phosphoproteome study identified 185 PME‐1‐regulated phosphopeptides in 128 proteins [36], indicating PME‐1‐mediated control of multiple cellular pathways and regulatory programs in cancer cells. Thereby, the anoikis sensitivity of PME‐1‐depleted cells is expected to be governed by several different mechanisms. To identify any such putative target mechanism, we chose to interrogate the impact of PME‐1 on mechanisms acknowledged to be involved in anoikis regulation [37, 38].

**Fig. 5:**
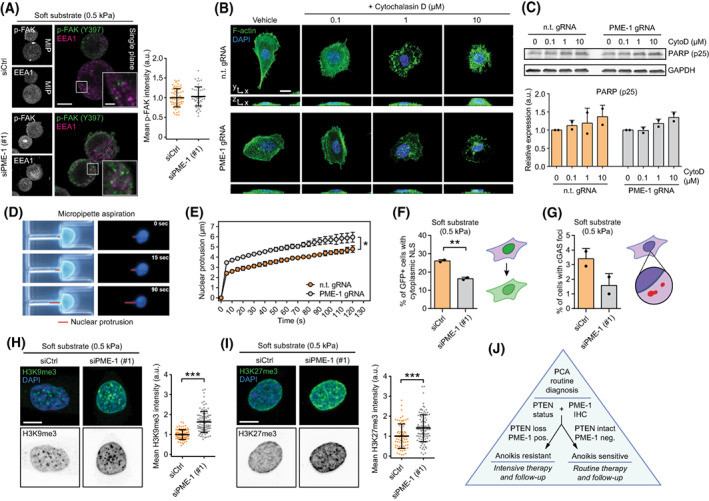
The potential mechanisms for PME‐1‐mediated anoikis resistance. (A) Immunofluorescence images (left) and quantification (right) depicting phosphorylated FAK (Y397) and early endosomal marker EEA1 in control and PME‐1‐depleted PC‐3 cells grown on soft (0.5 kPa) hydrogels for 24 h. Scale bar, 10 μm (main), 2 μm (inset). Mean ± SD from a representative of two independent experiments. (B) Fluorescence images depicting filamentous actin in control and PME‐1 KO PC‐3 cells treated with DMSO or increasing concentrations of Cytochalasin D (0.1–10 μm). Scale bar, 20 μm. Shown is a representative of two independent experiments. (C) Western blot (top) and quantification (bottom) displaying PARP cleavage in PC‐3 cells upon actin cytoskeleton disruption with Cytochalasin D. Mean ± SD of two independent experiments. (D, E) Images (D) and quantification (E) showing nuclear deformation in PC‐3 cells subjected to micropipette aspiration, as described in [46], over the course of 2 min. Mean ± SD of three independent experiments. *P < 0.05, Welch's *t*‐test. (F, G) Quantification of GFP‐NLS‐positive cells with cytoplasmic GFP signal (F) and the percentage of transfected cells with visible cGAS‐mCherry foci (G), respectively, in siCtrl‐ and siPME‐1‐treated cells grown on soft (0.5 kPa) substrate. Both readouts serve as a surrogate measure of compromised nuclear envelope. Mean ± SD of two independent experiments. **P < 0.01, Welch's *t*‐test (H–I). Immunofluorescence images and quantification depicting H3K9me3 (H) and H3K27me3 (I) levels in transiently PME‐1‐depleted and control PC‐3 cells, 48 h post‐transfection and 24 h after seeding on soft (0.5 kPa) substrate. Scale bar, 10 μm. Mean ± SD, representative of two independent experiments. ***P < 0.001, Mann–Whitney test. (J) Schematic representation of a putative model for including PME‐1 and its role in anoikis sensitivity/resistance in PCa diagnostics and therapy decision.

Related to the established role of focal adhesion kinase (FAK) signaling downstream of integrins in anoikis suppression [39], we performed an immunofluorescence analysis of phosphorylated FAK in control and PME‐1‐silenced cells that were grown on soft (0.5 kPa) hydrogels for 24 h. Even in low‐attachment conditions, endosomal FAK signaling can suppress anoikis and promote cell viability [18]. However, PME‐1 silencing did not affect the overall p‐FAK levels or existence of cytoplasmic p‐FAK foci in these same culture conditions which resulted in anoikis sensitization in PC‐3 cells (Figs 5A,B and 3D). Next, we analyzed the effects of PME‐1 silencing on the phosphorylation of pro‐survival kinase AKT and oncoprotein MYC by western blotting and by immunohistochemistry analysis of CAM tumours. However, no inhibition on either of these oncogenic and prosurvival signaling mechanisms were observed upon PME‐1 inhibition in either PC‐3 or DU‐145 cells (Fig. S3A–C). An additional candidate mechanism that could contribute to anoikis in PME‐1‐depleted cells was actin cytoskeleton remodeling upon cell rounding in low‐attachment conditions [37, 40]. To directly test whether disruption of the actin cytoskeleton would synergize with PME‐1 inhibition to induce apoptosis, control (n.t. gRNA) and PME‐1 KO (PME‐1 gRNA) cells were treated with increasing concentrations of cytochalasin D that prevents actin polymerization (Fig. 5B). However, western blotting analysis after 24 h of treatment with cytochalasin D did not show a PME‐1‐dependent difference in PARP cleavage, indicating that cell rounding and actin disruption are not causative mechanisms for inducing anoikis in PME‐1‐depleted cells (Fig. 5C).

To identify additional candidate mechanisms linked to PME‐1‐related anoikis regulation, we surveyed the most enriched cellular processes impacted by PME‐1 depletion based on the phosphoproteome data [36]. Interestingly, nuclear envelope assembly and chromatin remodeling were the two most significantly PME‐1‐regulated cellular processes (Table S3). Notably, PME‐1 physically associates with the nuclear lamina (NL) ([41] and Fig. S4A,B) and regulates not only phosphorylation of lamins, key structural components of the NL, but also other related proteins such as LAP2A/B that connect the NL to chromatin (Fig. S4C). We confirmed that nuclear lamins are increasingly dephosphorylated (detected with Lamin‐A/C phospho‐antibody) upon PME‐1 depletion (Fig. S4D,E) and that this effect is dependent on the PP2A complex B‐subunit PPP2R2A (Fig. S4D,E), an established tumour suppressor in PCa [6, 8]. Furthermore, NL directly influences chromatin architecture [42, 43], and NL de‐regulation can lead to DNA damage and cell death, limiting both cancer cell growth in soft agar and metastasis [42, 44, 45]. Therefore, we next assessed whether PME‐1 inhibition could alter nuclear structure and stability. To this end, nuclear deformability of control and PME‐1 KO cells was measured by subjecting them to a microfluidic micropipette aspiration experiment as previously described [46]. Notably, PME‐1 KO cells had significantly more deformable nuclei, implying structural differences (Fig. 5D,E). We then assessed whether these changes in nuclear mechanics would correlate with an increase in the number of harmful nuclear envelope ruptures [30] in PME‐1‐silenced cells. For this, we employed transient expression of GFP‐tagged nuclear localization signal (NLS), and mCherry‐tagged cyclic GMP–AMP synthase (cGAS), a cytosolic DNA sensor [30]. Surprisingly, PC‐3 cultures in low stiffness conditions displayed fewer cells with GFP‐positive cytoplasm and less cytoplasmic cGAS foci, indicative of fewer compromised nuclear envelopes, upon PME‐1 silencing (Fig. 5F,G and Fig. S5A–C). Together these results indicate that regardless of the newly identified role for PME‐1 in regulating NL proteins (Fig. S4C) and nuclear mechanics (Fig. 5D,E), the anoikis sensitivity of PME‐1‐depleted PC‐3 cells cannot be explained directly by apoptosis‐inducing nuclear envelope disruptions.

However, PME‐1‐mediated alterations in different NL proteins (Fig. S4C) could also impact cell viability indirectly, by regulating chromatin structure and transcription (Table S3). Chromatin condensation is dependent on intact NL, and heterochromatin structures are linked to the lamina via trimethylated histone H3 (H3K9me3 and H3K27me3) [43, 47, 48]. On the other hand, increased H3K27me3 and H3K9me3 levels sensitize cancer cells to apoptosis [22, 23, 49]. Thus, we studied whether PME‐1 depletion would affect H3K9me3 and H3K27me3 levels in PC‐3 cells grown on soft hydrogels. Indeed, PME‐1 depletion was found to increase both H3K9me3 and H3K27me3 levels on soft substrate, concordant with the increased apoptosis observed in similar conditions (Figs 5H,I and 3D). However, consistent with great heterogeneity in PCa and our model that PME‐1‐mediated phosphoproteome regulation can control different anoikis sensitivity mechanisms, we did not see evidence for increased histone 3 trimethylation in DU‐145 cells upon PME‐1 depletion on soft substrate (data not shown). Thereby, an increase in histone H3 methylation, and its subsequent apoptosis promoting effects [22, 23, 49], may contribute to the increased anoikis sensitivity in some PME‐1‐depleted PCa cells, whereas in others alternative mechanisms are at play. However, the direct mechanistic link between PME‐1 and histone H3 methylation remains to be identified.

## 4 Discussion

Here, we discover a clinically relevant role for the PP2A methylesterase PME‐1 in supporting anchorage‐independent growth of PCa cells with compromised PTEN. Our data indicate that the anchorage‐independence of PCa cells with concomitant inhibition of the two tumour suppressor phosphatases, PP2A and PTEN [3, 9], can largely be explained by their resistance to anoikis. Although anoikis suppression is a generally relevant mechanisms for tumour progression [15, 37], it may be of particular clinical importance in slowly progressing cancers such as PCa, where tumours can be diagnosed in the indolent phase, and there is a strong need to be able to both predict and inhibit the likelihood of disease progression [2].

Integrins act as key survival signaling receptors suppressing anoikis [19]. Here, we demonstrate that the onset of anoikis in PCa requires concomitant loss of both integrin‐mediated matrix rigidity‐dependent signaling and PME‐1‐mediated PP2A inhibition. Given the complexity of both signaling programs, the contributing molecular mechanisms are expected to be numerous, impeding the identification of a single key mechanism. Nevertheless, we could exclude several classical anoikis‐related mechanisms from being involved in the increased anoikis sensitivity of PME‐1‐inhibited PCa cells with relative confidence. On the other hand, our results demonstrating a significant increase in apoptosis‐promoting H3K9 and H3K27 trimethylation [22, 23, 49] at least in PC‐3 cells do provide an interesting clue for the underlying mechanism relevant in some PCa cells. Although the direct mechanistic link between PME‐1 and histone H3 methylation remains to be addressed in future studies, both the phoshoproteome data and increased nuclear deformability suggest that it could be attributed to abnormal NL‐chromatin mechanics in the PME‐1‐deficient PCa cells. The abnormal NL‐chromatin mechanics could also lead to other anoikis sensitizing changes beside histone 3 trimethylation, in other PME‐1 depletion sensitive PCa cells. Collectively our results demonstrate a role for PME‐1‐mediated PP2A inhibition in protecting several *PTEN*‐deficient cell models from anoikis through divergent downstream mechanisms.

PTEN and PP2A have both been identified independently as PCa tumour suppressor phosphatases [3, 4, 6, 7, 8], but the clinical relevance of their co‐operation has not been studied thus far. *PTEN* deficiency promotes anoikis resistance of PCa cells [50] and our data indicate that concomitant inhibition of a second tumour suppressor phosphatase PP2A by PME‐1 renders the *PTEN*‐deficient cells particularly well protected from anoikis. We hypothesize that this is likely to contribute to the observed clinical aggressiveness of these cancers. Mechanistically, PTEN‐mediated anoikis resistance is mediated by AKT signaling [50, 51], whereas we observed that PME‐1 depletion had no consistent effect on AKT phosphorylation (Fig. S3A,B). This indicates non‐overlapping downstream mechanisms for PTEN and PME‐1 in anoikis suppression, further explaining their synergetic actions. As *PTEN* genetic status can be routinely evaluated in current clinical PCa diagnostic practice [2], our results indicate a diagnostic utility of assessment of PME‐1 status for patients with complete *PTEN* loss. Definition of PME‐1 protein expression as a surrogate for PP2A activity status in tissues samples would greatly simplify biomarker analysis of PP2A function because it sidesteps the need for analyzing all the possibly relevant subunits. Moreover, as PME‐1 inhibits directly PP2A‐C catalytic activity, in addition to regulating its C‐terminal tail methylation [10, 14], analysis of PME‐1 expression could be a superior diagnostic method over detection of the methylation status of the PP2A‐C by modification sensitive antibodies. Although further studies are clearly needed to validate these conclusions, our results indicate that patients with *PTEN* ^loss^/PME‐1^high^ tumours might benefit from more intensive follow‐up, and/or from more aggressive therapies as first, and second line treatments (Fig. 5J).

Together these results identify anoikis resistance as a candidate mechanism by which PME‐1‐mediated PP2A inhibition promotes malignant progression of *PTEN*‐deficient PCa. Together with emerging orally bioavailable PP2A reactivating compounds, exhibiting profound antitumour activity in *in vivo* PCa models [52], these results clearly emphasize the future importance of comprehensive understanding of PP2A biology for management of aggressive PCa. Future studies would also be needed to validate whether the functional co‐operation of PTEN and PP2A extends to other cancers beyond PCa, and whether these findings would open novel therapeutic opportunities for simultaneously targeting both of these tumour suppressor phosphatases [53].

## 5 Conclusions

PTEN and PP2A are both tumour suppressor phosphatases, but the clinical cancer relevance of their co‐operation has not been studied thus far. These results demonstrate that concomitant loss of activity of PTEN and PP2A is associated with particularly aggressive PCa phenotype and with early patient relapse from standard therapy. In preclinical cell and animal models double PTEN‐PP2A inhibition potently suppressed anoikis‐type apoptosis induction. PP2A inhibition in these models was mediated by overexpression of its methylesterase PME‐1 which, based on these results could constitute a potential biomarker for evaluating aggressivity of PCas with PTEN loss. Future research is however needed to fully understand how PME‐1 overexpression mechanistically leads to anoikis resistance in PTEN‐deficient PCa cells.

## Conflict of interest

The authors declare no conflict of interest.

## Author contributions

Conceived and designed the project; AA, CR, YRP, LT, PT, JL, JI, JW. Acquired the data; AI, PS, AK, AA, CR, AE, NG, OK, AP, AK, SPL, IP. Analyzed and interpreted the data; AI, PS, AK, AA, CR, NG AE, AK, AR, IP, TM. Wrote the paper; AI, AA, AE, JI, JW.

### Peer review

The peer review history for this article is available at https://publons.com/publon/10.1002/1878‐0261.13353.

## Supporting information


**Fig. S1.** High PME‐1 expression associates with total PTEN loss in prostate cancer patient samples.
**Fig. S2.** Modulation of PC‐3‐integrin ligand interaction using biotinylated PLL‐g‐PEG and streptavidin‐conjugated fibronectin fragment.
**Fig. S3.** PME‐1 inhibition does not inhibit AKT or MYC signaling in prostate cancer cells.
**Fig. S4.** PME‐1 co‐localizes with Lamin‐A/C and regulates the phosphorylation of multiple nuclear lamina components.
**Fig. S5.** PME‐1 silencing does not compromise PC‐3 nuclear envelope integrity on soft substrates.

**Table S1.** Demographics of the radical prostatectomy patient cohort.

**Table S2.** Comparison of benign and cancer PME status.

**Table S3.** Enrichment of cellular processes based on PME‐1 regulated phosphopeptides.

## Data Availability

Data and research materials are available upon reasonable request by contacting the corresponding author.
