# Supplementary material for: PP2A methylesterase PME‐1 suppresses anoikis and is associated with therapy relapse of PTEN ‐deficient prostate cancers
Source: Mol Oncol. 2023 Apr 18;17(6):1007–23. doi: 10.1002/1878-0261.13353 (PMC10257411; doi:10.1002/1878-0261.13353)
Supplement: Supplementary file 1 — Fig. S1. High PME‐1 expression associates with total PTEN loss in prostate cancer patient samples. Fig. S2. Modulation of PC‐3‐integrin ligand interaction using biotinylated PLL‐g‐PEG and streptavidin‐conjugated fibronectin fragment. Fig. S3. PME‐1 inhibition does not inhibit AKT or MYC signaling in prostate cancer cells. Fig. S4. PME‐1 co‐localizes with Lamin‐A/C and regulates the phosphorylation of multiple nuclear lamina components. Fig. S5. PME‐1 silencing does not compromise PC‐3 nuclear envelope integrity on soft substrates. [file MOL2-17-1007-s002.pdf]

A

PME-1 vs PTEN Status

	PME-1 Low	PME-1 High	Total	P
PTEN Intact	193 (81.1)	57 (69.5)	250	**0.043**[a]
Complete PTEN Loss	45 (18.9)	25 (30.5)	70	
Total	238	82	320	

[a] Fisher's exact test

PME-1 vs AR Status

	PME-1 Low	PME-1 High	Total	P
AR Low	97 (40.8)	14 (17.1)	111	**< 0.001**[a]
AR High	141 (59.2)	68 (82.9)	209	
Total	238	82	320	

PME-1 vs ERG Status

	PME-1 Low	PME-1 High	Total	P
ERG Negative	128 (53.8)	27 (32.9)	155	**0.001**[a]
ERG Positive	110 (46.2)	55 (67.1)	165	
Total	238	82	320	

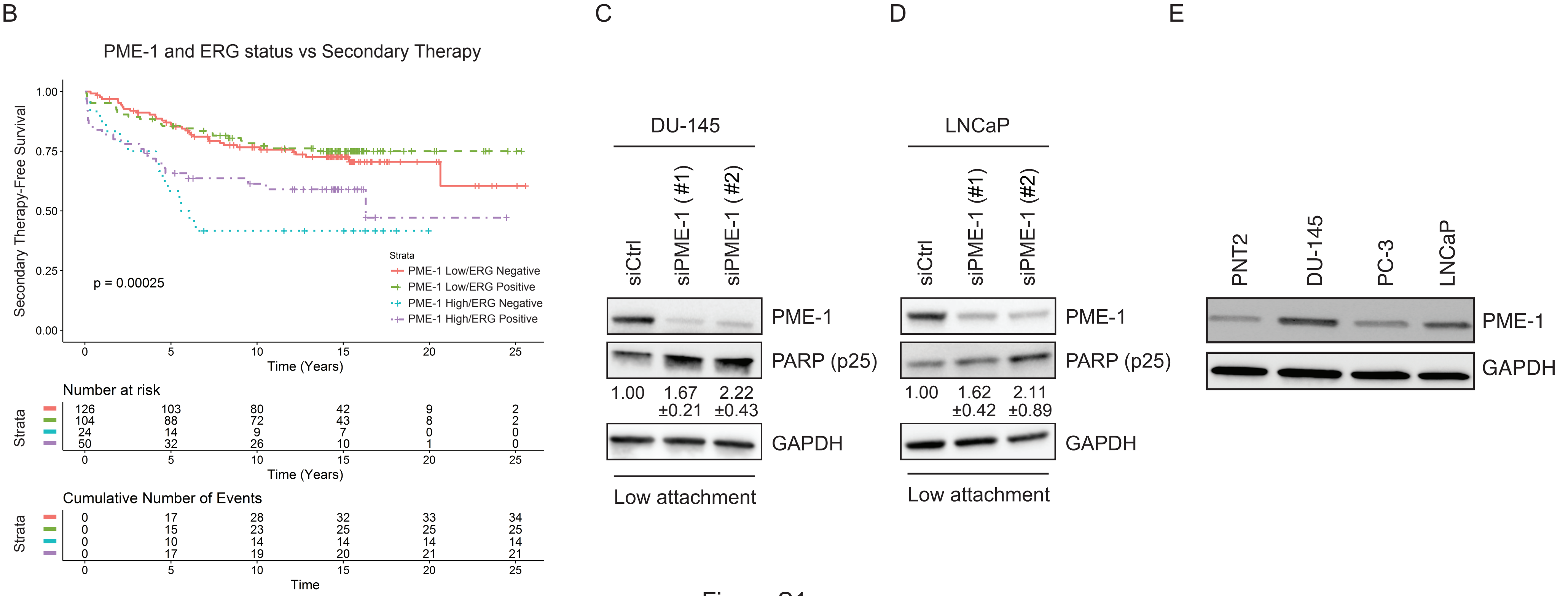

Figure S1

Figure S1. High PME-1 expression associates with total PTEN loss in prostate cancer patient samples

A. PME-1 status was correlated to previously assessed PTEN, AR and ERG status. PME-1 expression significantly associate with complete PTEN loss, but also with high AR expression and ERG positivity status, as analysed by Fisher's exact test. B. Kaplan-Meyer analysis of time to secondary therapies after primary treatment, based on PME-1 status in combination with ERG. C,D. siCtrl- and siPME-1-transfected DU-145 (C) or LNCaP (D) cells were plated 72 h post-transfection on low attachment plates for 24 h, before collection and lysis, and subsequently analyzed by western blotting for cleaved PARP-1 (PARP (p25)). A representative blot is shown. Numbers below the PARP lane indicate mean PARP/GAPDH fold change based on three independent biological repeats. E. PME-1 protein expression in indicated prostate cell lines, where PNT2 is a normal prostate epithelial cell line immortalized with SV40 virus.

PME-1, Protein phosphatase methylesterase 1; PTEN, Phosphatase and Tensin homolog; AR, Androgen receptor; ERG, ETS transcription factor ERG; siCtrl, control siRNA; siPME-1, PME-1 siRNA; siRNA; Small interfering RNA; PARP, Poly(ADP-ribose) polymerase; GAPDH, Glyceraldehyde-3-phosphate dehydrogenase

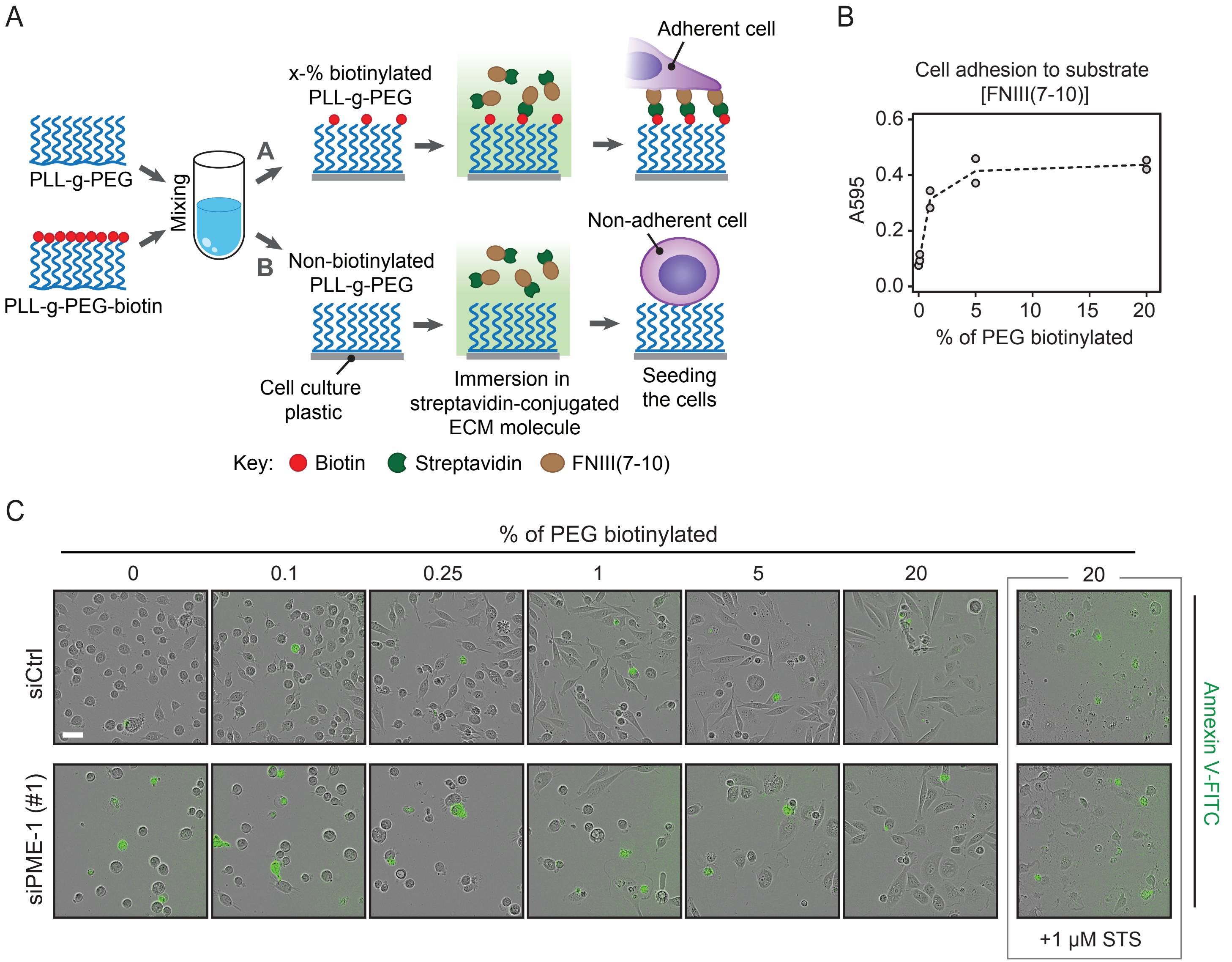

Figure S2

Figure S2. Modulation of PC-3-integrin ligand interaction using biotinylated PLL-g-PEG and streptavidin-conjugated fibronectin fragment.
A. Schematic representation of the preparation of PLL-g-PEG-coated tissue culture surfaces. Stock solutions of PLL-g-PEG and PLL-g-PEG-biotin were mixed together in 10 mM Hepes buffer (pH 7.4) to yield PLL-g-PEG-solutions with varying amounts of biotinylated compound (A), or no biotin groups at all (B). Next, biotinylated PLL-g-PEG was coupled to streptavidin-conjugated FNIII(7-10) to enable selective adhesion of cells to specific coated surfaces. B. Standard curve depicting PC-3 adhesion to PLL-g-PEG- and FNIII(7-10)-coated plastic over 30 min. Cell-substrate adhesion increases as a function of the fraction of biotinylated PEG. C. Phase contrast images and overlaid fluorescence data showing control and PME-1-depleted PC-3 cells, as well as Annexin V positive cells and debris after 60 h on PLL-g-PEG- and FNIII(7-10)-coated plastic. Scale bar, 50 μm.
PLL-g-PEG, Poly(l-lysine)-g-poly(ethylene glycol); PEG, Poly(ethylene glycol); STS, Staurosporine

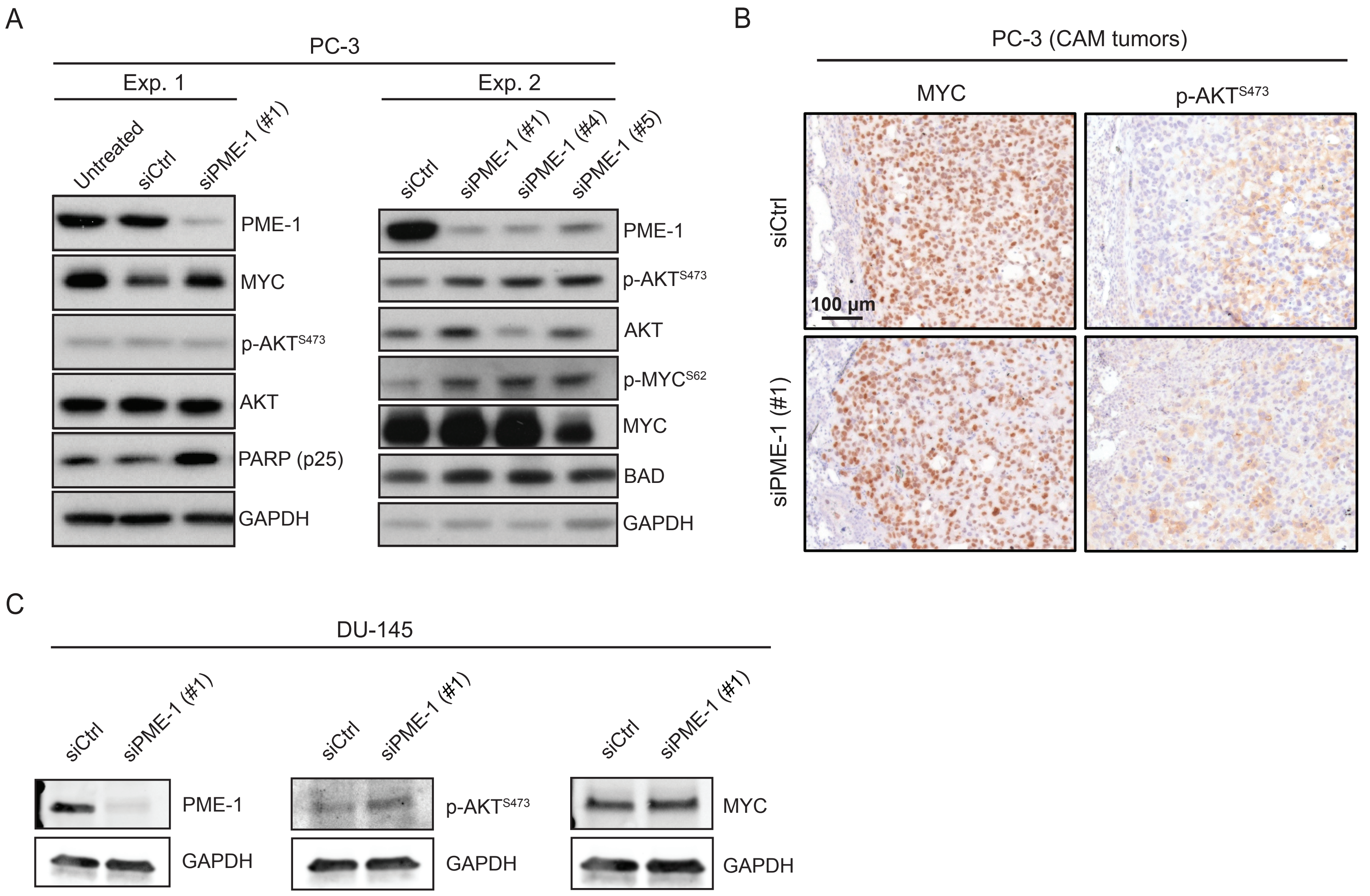

Figure S3

Figure S3. PME-1 inhibition does not inhibit AKT or MYC signaling in prostate cancer cells
A. The effect of PME-1 depletion on AKT and MYC in PC-3 cells was analysed by western blotting. B. The effect of PME-1 silencing on MYC levels and AKT phosphorylation was assayed by immunohistochemistry of PC-3 CAM tumors. Scale bar 100 uM for all panels.
C. The effect of PME-1 depletion on AKT and MYC in DU-145 cells was analysed by western blotting.
CAM, Chick chorioallantoic membrane; siCtrl, control siRNA; siPME-1, PME-1 siRNA; siRNA; Small interfering RNA; AKT, AKT serine/threonine kinase 1; pAKT, phosphorylated AKT; MYC, MYC proto-oncogene; pMYC, phosphorylated MYC; BAD, BCL2 associated agonist of cell death PARP, Poly(ADP-ribose) polymerase; GAPDH, Glyceraldehyde-3-phosphate dehydrogenase

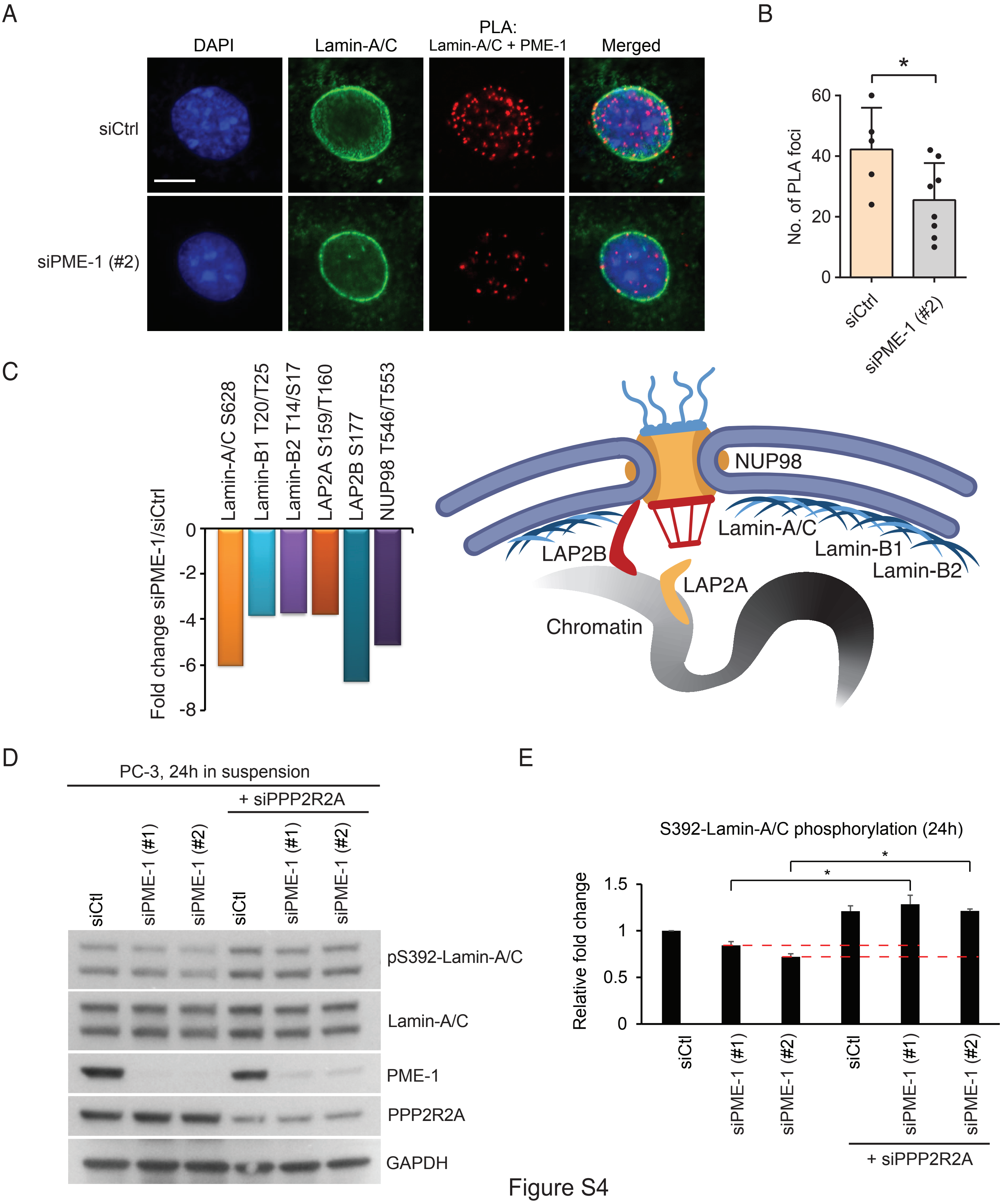

Figure S4

Figure S4. PME-1 co-localizes with Lamin-A/C and regulates the phosphorylation of multiple nuclear lamina components. A. Proximity ligation assay and immunofluorescence illustrate the co-localization of PME-1 with Lamin-A/C in siRNA-treated PC-3 cells. DNA was counterstained using DAPI. Scale bar, 10 µm. B. The number of PLA foci in siCtrl- and siPME-1-transfected cells. Mean ± SD, *$p < 0.05$, Mann-Whitney test. C. PME-1 was found to regulate the phosphorylation of lamins, LAP2A/B and NUP98. The graph is based on phosphoproteomics data published in Kauko et al., 2020 (Ref. 32). D. To show the PP2A-dependence of Lamin-A/C phosphorylation regulation by PME-1, PC-3 cells were transiently co-depleted of PME-1 and PP2A B-subunit PPP2R2A. After transfection, cells were cultured on low attachment plates for 24h, prior to sample collection, lysis and subsequent analysis of p-Lamin-A/C PME-1 and PPP2R2A. E. Relative change in pS392-Lamin-A/C signal as compared to total Lamin-A/C in PME-1 depleted cells. Expression in siCtrl transfected cells was set as fold 1. * $p<0.05$ t-test.
DAPI, 4′,6-diamidino-2-phenylindole; PLA, Proximity ligation assay; siCtrl, control siRNA; siPME-1, PME-1 siRNA; siRNA; Small interfering RNA; PME-1, Protein phosphatase methylesterase 1; LAP2A/B, Lamina-associated polypeptide 2 alpha/beta; NUP98, Nucleoporin 98; PPP2R2A, PP2A B-subunit (B55alpha)

A

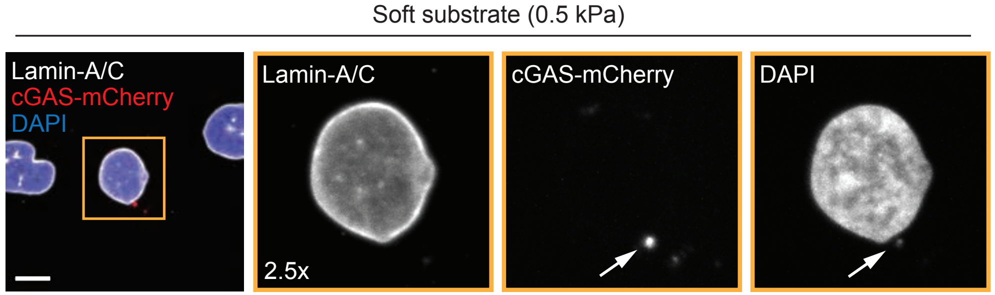

B

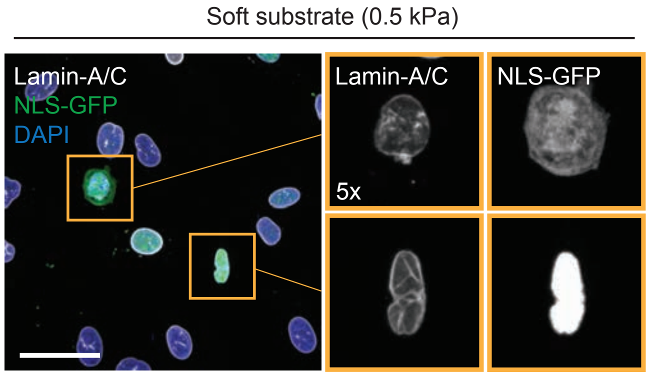

C

siRNA	NLS-GFP cytoplasmic (%)		p-value (Chi-squared)
	–	+	
Ctrl	152 (74.1)	53 (25.9)	
PME-1	155 (83.8)	30 (16.2)	**0.020**

siRNA	cGAS-mCherry foci (%)		p-value (Chi-squared)
	–	+	
Ctrl	1131 (96.7)	38 (3.3)	
PME-1	1009 (98.5)	15 (1.5)	**0.007**

Figure S5

Figure S5. PME-1 silencing does not compromise PC-3 nuclear envelope integrity on soft substrates.
A. Immunofluorescence image depicting Lamin-A/C, cytoplasmic DNA and a corresponding cGAS-mCherry aggregate (white arrows) in a siCtrl-transfected PC-3 cell on soft (0.5 kPa) hydrogel. Scale bar, 10 μm. B. Immunofluorescence image showing Lamin-A/C and NLS-GFP in siCtrl-transfected PC-3 cells on soft (0.5 kPa) hydrogel. ROI: some of the cells present with cytoplasmic NLS-GFP (top), implying a compromised nuclear envelope, while in others the protein is constrained solely in the nucleus (bottom). Scale bar, 50 μm. C. Contingency tables depicting all the GFP positive cells analyzed for NLS localization (top) and all the transfected cells analyzed for the presence of cGAS-mCherry foci (bottom).
Pooled results from two independent experiments.
