# Supplementary material for: PP2A methylesterase PME‐1 suppresses anoikis and is associated with therapy relapse of PTEN ‐deficient prostate cancers
Source: Mol Oncol. 2023 Apr 18;17(6):1007–23. doi: 10.1002/1878-0261.13353 (PMC10257411; doi:10.1002/1878-0261.13353)
Supplement: Supplementary file 3 — Table S2. Comparison of benign and cancer PME status. [file MOL2-17-1007-s004.pdf]

Supplementary Table 2 - Comparison of Benign and Cancer PME status.

	**Benign - PME Low**	**Benign - PME High**
Cancer - PME Low	219	2
Cancer - PME High	73	3
Total	292 (98.6%)	5 (1.7%)

	Benign - Negative	Benign - Low	Benign - Intermediate	Benign - High
Cancer - Negative	0	1	1	0
Cancer - Low	5	14	16	0
Cancer - Intermediate	12	76	94	2
Cancer - High	5	38	30	3
Total	22	129	141	5
