# Supplementary material for: PP2A methylesterase PME‐1 suppresses anoikis and is associated with therapy relapse of PTEN ‐deficient prostate cancers
Source: Mol Oncol. 2023 Apr 18;17(6):1007–23. doi: 10.1002/1878-0261.13353 (PMC10257411; doi:10.1002/1878-0261.13353)
Supplement: Supplementary file 4 — Table S3. Enrichment of cellular processes based on PME‐1 regulated phosphopeptides. [file MOL2-17-1007-s001.pdf]

Table S3. Enrichment of cellular processes based on PME-1 regulated phosphopeptides

GO.ID	Description	p.Val	FDR
GO:0006338	Chromatin remodeling	0.0425	0.0425
GO:0045103	Intermediate filament-based process	0.00192	0.00192
GO:0045104	Intermediate filament cytoskeleton organization	0.00177	0.00177
GO:0071103	DNA conformation change	0.00596	0.00596
GO:0006265	DNA topological change	0.00769	0.00769
GO:0033184	Positive regulation of histone ubiquitination	0.0137	0.0137
GO:0006997	Nucleus organization	0.0441	0.0441
GO:0031468	Nuclear envelope reassembly	0.00351	0.00351
GO:1903312	Negative regulation of mRNA metabolic process	0.0285	0.0285
GO:0006397	mRNA processing	0.0493	0.0493
GO:0050684	Regulation of mRNA processing	0.0288	0.0288
GO:0050686	Negative regulation of mRNA processing	0.013	0.013
GO:0006921	Cellular component disassembly involved in execution phase of apoptosis	0.0211	0.0211
GO:0043414	Macromolecule methylation	0.0161	0.0161
REAC:2980766	Nuclear Envelope Breakdown	0.00239	0.00239
REAC:4419969	Depolymerisation of the Nuclear Lamina	0.000472	0.000472
REAC:2993913	Clearance of Nuclear Envelope Membranes from Chromatin	0.0315	0.0315
REAC:2995410	Nuclear Envelope Reassembly	0.000209	0.000209
REAC:2995383	Initiation of Nuclear Envelope Reformation	0.000209	0.000209
